# Effects of head trauma and sport participation in young-onset Parkinson’s disease

**DOI:** 10.1007/s00702-021-02370-8

**Published:** 2021-07-14

**Authors:** Tommaso Schirinzi, Piergiorgio Grillo, Giulia Di Lazzaro, Henri Zenuni, Chiara Salimei, Kristen Dams-O’Connor, Giulia Maria Sancesario, Nicola Biagio Mercuri, Antonio Pisani

**Affiliations:** 1 Department of Systems Medicine, Unit of Neurology, University of Roma Tor Vergata, Via Montpellier, 00133 Rome, Italy; 2 Neurology Unit, IRCCS Fondazione Policlinico Universitario A. Gemelli, Rome, Italy; 3 Department of Rehabilitation and Human Performance, Department of Neurology, Icahn School of Medicine at Mount Sinai, New York, USA; 4 Biobank Unit, IRCCS Fondazione Santa Lucia, Rome, Italy; 5 Experimental Neuroscience Unit, IRCCS Fondazione Santa Lucia, Rome, Italy; 6 Department of Brain and Behavioral Sciences, University of Pavia, Pavia, Italy; 7 IRCCS Mondino Foundation, Pavia, Italy

**Keywords:** Head trauma, TBI, Young-onset Parkinson’s disease, Sport, Tau

## Abstract

Head trauma (HT) is emerging as an event anticipating onset of neurodegenerative disorders. However, the potential contribution of HT in young-onset cases (YOPD, age at onset < 50) of Parkinson’s disease (PD) has not been examined yet. Here, we systematically assessed HT history in PD patients to estimate the risk associated, especially in terms of age of onset, and define the correlations with the clinical-biochemical profile. The Brain Injury Screening Questionnaire (BISQ) was administered to 94 PD patients (31 with YOPD, known monogenic forms excluded) and 70 controls. HT history was correlated with motor and non-motor scores in all patients, and to CSF biomarkers of neurodegeneration (α-synuclein, amyloid-β42, total and phosporiled-181 tau, lactate, CSF/serum albumin) into a subgroup. HT increased the risk for both PD and YOPD. In PD patients, but not in those with YOPD, the number of HTs directly correlated with CSF total-tau levels. No other correlations resulted between HT and clinical parameters. Sport-related HT was a specific risk factor for YOPD; conversely, the prolonged sporting life represented a protective factor. HTs can favor PD onset, even as YOPD. Sport-related HT resulted a risk factor for YOPD, although the longer sporting practice delayed PD onset, protecting from YOPD. Tauopathy may underlie the overall association between HT and PD. Additional mechanisms could be instead implicated in HT contribution to YOPD onset.

## Introduction

Parkinson’s disease (PD) is a disabling neurodegenerative disorder, with idiopathic and multifactorial origin (Kalia and Lang 2015; Surmeier et al. 2017; Petrillo et al. 2019). Common risk factors for the classical presentation of PD in elderly include aging, toxicants exposure, and cardiovascular diseases (Ascherio and Schwarzschild 2016; Imbriani et al. 2020). However, more rarely, PD may appear before the age of 50, as “young onset PD” (YOPD), in the absence of apparently known risk factors (Ylikotila et al. 2015; Mehanna and Jankovic 2019; Schirinzi et al. 2020b). In animal models of amyotrophic lateral sclerosis (ALS), head injury precipitates genetic susceptibility and anticipates clinical-pathological onset of the disease (Alkaslasi et al. 2021). As well, people with head traumas may develop young-onset dementia (Nordstrom et al. 2014; LoBue et al. 2017; Lobue and Cullum 2019). Similarly, it might be hypothesized that head trauma (HT) can contribute to accelerate PD onset in those individuals with genetic vulnerability, thus representing a potential risk factor for YOPD.

Several studies revealed the link between head trauma and PD (Jafari et al. 2013; Ascherio and Schwarzschild 2016; Crane et al. 2016; Nicoletti et al. 2017), although with controversial results, different experimental approaches (Kenborg et al. 2015; Hasan et al. 2020), and limitations due to imprecise or incomplete ascertainment of trauma exposure. Moreover, prior investigations mostly focused on more severe traumatic brain injury (TBI), such as injuries resulting in loss of consciousness, despite evidence that even milder and subclinical HTs can be associated with PD and other neurodegenerative diseases (Dams-O’Connor et al. 2014; Crane et al. 2016; Alosco et al. 2018; Gardner et al. 2018). Consequently, the nature of the relationship between HT and PD needs to be further elucidated by a systematic evaluation of HT history in PD patients, including those with YOPD. As well, circumstance-related HT, such as sporting activities, have to be determined to identify possible preventive interventions.

The retrospective examination of lifetime HT, including remote injuries, can be challenging and complex; however, structured self-reported screening tools, including the Brain Injury Screening Questionnaire (BISQ), have recently been developed to reliably characterize HT history across the lifespan (Dams-O’Connor et al. 2014).

In this study, we used the BISQ to assess and characterize HT history in a cohort of PD patients to estimate the associated risk, the impact on age of onset, and the effect on main clinical features of the disease. In addition, since previous studies on HT and PD mostly relied on clinical diagnoses or medical claims data in the absence of biological markers (Gardner et al. 2015), we measured, in a subset of patients, the correlations between HT and CSF biomarkers of neurodegeneration (Schirinzi et al. 2018a, 2021; Sancesario et al. 2020), such to better clarify the role of HT in PD, and particularly in YOPD.

## Methods

### Subjects

The study involved a total of 164 subjects, 94 PD patients and 70 healthy controls, enrolled at the Neurology Unit of Tor Vergata University Hospital (Rome—Italy; 2019–2020). PD was diagnosed according to 2015 MDS criteria (Postuma et al. 2015); controls were sex/age-matched healthy subjects (volunteers and non-blood relatives of patients), without neurological disorders.

All participants were screened for HT history using the Brain Injury Screening Questionnaire (BISQ) (Dams-O’Connor et al. 2014), In brief, BISQ systematically characterizes lifetime exposure to head trauma by providing a list of events that may result in head trauma (e.g., during different sports, military service, motor vehicle accidents, or other activities/accidents) to facilitate recall of injuries, and for each blow to the head reported, subsequent queries permit estimates of TBI severity (e.g., presence of altered mental status or unconsciousness). In the current study, those in the PD group were asked to report lifetime head trauma preceding diagnosis.

The following variables were created from the BISQ: trauma score (TS), which counted the total number of blows to the head (HTs) per person, regardless of the cause or the severity; HT history, classified as positive (Tr +) if TS ≥ 1, or negative (Tr−) if TS = 0; circumstances of HT, divided in sport-related or non-sport related (any other etiologies, such as motor vehicle accident, assault, fall, etc.); classification of HT as simple blow (no consciousness impairment) or traumatic brain injury (TBI) (any mental state alteration or any loss of consciousness (Ruff et al. 2009); history of sport participation, including team sports, (e.g. soccer, volley), water sports (e.g. swimming), individual sports (e.g. tennis), athletics/gymnastics/running, contact sports (e.g., boxing, combat sports), other sports; and total years of sport practice (summing the years of participation in each activity). Data regarding military activity were not considered in the analysis because of the small number of those who reported military service. Medical history and demographics were recorded.

In PD patients, age at onset, disease duration, Unified Parkinson Disease Rating Scale part 3 (UPDRSIII), Hoehn and Yahr stage (H&Y), Non Motor Symptoms Scale (NMSS), Mini Mental State Examination (MMSE) adjusted for age and educational level, and the levodopa equivalent daily dose (LEDD) were collected in concomitance with questionnaire (assessment obtained in “on” state, under the effects of antiparkinsonian medications). As previously described (Schirinzi et al. 2020b), PD patients were grouped depending on the age at onset in young onset (YOPD: onset ≤ 50 years; *n* = 31; monogenic cases, namely *SNCA, LRRK2, PRKN, PINK1, DJ-1* and *GBA* were excluded) and late onset (LOPD: onset > 50 years; *n* = 63); none had juvenile-onset (< 21 years) (Mehanna and Jankovic 2019).

A panel of CSF biomarkers including α-synuclein (α-syn), amyloid-β42 (Aβ42), total tau (t-tau), phosporiled-181-tau (p-tau), lactate, CSF/serum albumin ratio (AR) was available for 24 patients (YOPD *n* = 13; LOPD *n* = 11). In these subjects, lumbar puncture and biomarkers assay were conducted as previously reported (Schirinzi et al. 2018b; Sancesario et al. 2020). The biomarkers Aβ42, t-tau and p-tau were quantified by a two-step sandwich chemiluminescent enzyme-immunoassay (CLEIA) method on a Lumipulse G600II (Fujirebio Diagnostics) following standard procedures. Intra- and inter-assay coefficients of variation (CVs) assessed in CSF samples and internal standards (low, medium, and high concentrations of each parameter) were lower than 10%. Level of α-syn was assessed using Human α-Synuclein Enzyme immunoassay (ELISA) Kit (Biolegend), with intra- and inter-assay CVs lower than 20%.

The study was conducted in agreement with ethical principles of Helsinki declaration, after the approval of the local committee (protocol number 0026092/2017). All participants signed an informed consent.

### Statistical analysis

Data distribution was evaluated with the Shapiro–Wilk test. Continuous variables were compared between groups with parametric (one-way ANOVA) or non-parametric tests (Mann–Whitney *U* test), as appropriate. Categorical variables were compared by chi-square test.

To evaluate the association between HT (either as TS or Tr + /Tr− condition) and clinical groups (PD vs controls, YOPD vs LOPD), binomial logistic regressions were performed. Correlations between TS and clinical parameters or biomarkers were tested by liner regression, either as simple model or adjusted for main covariates (age, sex, disease duration, and LEDD when appropriate). As well, clinical parameters and biomarkers were compared between Tr + and Tr− patients by both one-way ANOVA and one-way ANCOVA (adjusted for age, sex, disease duration, and LEDD when appropriate).

Statistical significance was set at *p* < 0.05. Statistical analysis was performed in blind using IBM-SPSS-22. Data are available from authors upon reasonable request.

## Results

Table 1 summarizes main features of the study population.

**Table 1:** Clinical and demographic of the study population

	Control		PD		Significance	YOPD		LOPD		Significance
**Clinical variable**	*n* = 70		*n* = 94			*n* = 31		*n* = 63		
Sex (%m/f)	60/40		47/53		ns	59/61		61/59		ns
	Mean	St.dev	Mean	St.dev		Mean	St.dev	Mean	St.dev	
Age (y)	60.7	11.6	62.8	11	ns	50.7	6.9	69.1	7	***p* < 0.001**
TS	0.8	1.4	1.2	1.3	***p* = 0.002**	1.3	1	1.2	1.5	ns
Age at onset (y)	–		58.9	11.3	–	45.7	5.2	65.6	6.8	***p* < 0.001**
Disease duration (y)	–		4.1	3.6	–	4.3	4.3	4	3.3	ns
H&Y	–		1.9	0.5	–	1.8	0.6	2	0.5	ns
UPDRS III	–		18.7	8	–	18.5	7.8	19.3	8.1	ns
MMSE	–		28.1	2.1	–	28.8	1.7	27.6	2.3	ns
NMSS	–		41.8	35	–	38.1	32	44.1	38.1	ns
LEDD	–		348.4	373.3	–	389.5	405.5	328.9	353	ns
**CSF biomarkers**			*n* = 24			*n* = 13		n = 11		
	Mean	St.dev	Mean	St.dev		Mean	St.dev	Mean	St.dev	
α-syn (pg/ml)	–		460	339.9	–	422.5	260.5	400.4	490.4	ns
Aβ42 (pg/ml)	–		829.8	250.2	–	854.7	245.6	810.7	279.3	ns
t-tau (pg/ml)	-		184.3	122.5	–	143.6	73.4	243	156.8	***p* = 0.05**
p-tau (pg/ml)	–		34.3	14.2	–	29.7	10.1	40.1	17.7	ns
Lactate (mmol/l)	–		1.6	0.4	–	1.7	0.4	1.5	0.4	ns
AR	–		12.1	17.5	–	15.5	23.2	8.4	5.1	ns

y = years; m = male; f = female; other abbreviations are spelled out in the text

Bold values indicate statistical significance

### Head trauma and PD

**Fig. 1:**
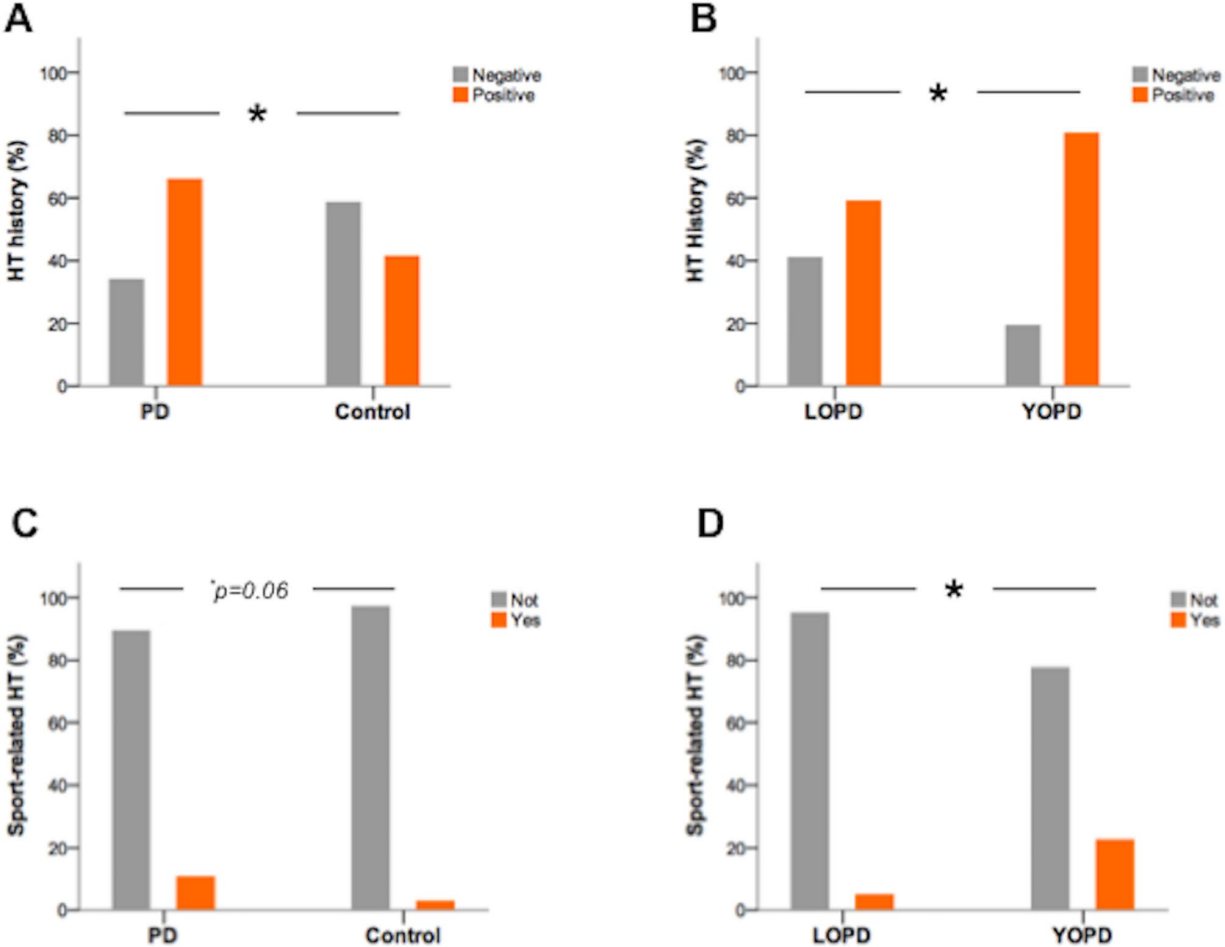
Prevalence of HT history (Tr + and Tr−) in PD vs controls (**A**) and YOPD vs LOPD (**B**). Prevalence of sport-related HT in PD vs controls (**C**) and YOPD vs LOPD (**D**). Asterisks indicate statistical significance (*p* < 0.05) obtained by chi-square test

The percentage of subjects with positive HT history (Tr +) was significantly higher in PD than controls (66% vs 41%, *p* = 0.002%), in the absence of gender differences (Fig. 1). Individuals Tr + had an increased risk for PD (OR 2.7, 95% CI 1.4–5.2; *p* = 0.002).

Trauma score (TS) was significantly higher in PD (median = 1; mean ± st.dev. = 1.23 ± 1.33) than controls (median = 0; mean ± st.dev. = 0.76 ± 1.4) (*U* = 2431, *p* = 0.002). No gender differences existed in either group. Binomial logistic regression indicated that TS increased the risk for PD (Odd Ratio, OR 1.36, 95% CI 1.02–1.8; *p* = 0.03).

Head blows (trauma without consciousness impairment) constituted 65% of HTs reported in PD patients and 77% in controls (*p* = not significant, NS). Regarding HTs with consciousness impairment (TBIs), they occurred in 35.4% of PD patients and 23% of controls (*p* = NS). Specifically, TBI with only altered mental status occurred in 26.6% of PD patients and 17.1% of control (*p* = NS). TBI with loss of consciousness occurred in 25.5% of PD patients and 11.4% of controls (*p* < 0.05), representing a risk factor for PD (OR 2.65, 95% CI 1.1–6.3; *p* = 0.03).

HT occurred during sport practice in 10.6% of PD patients and 2.9% of controls (*p* = 0.06), during non-sport activities in 62.8% of patients and 41.4% of controls (*p* = 0.007). Binomial logistic regression indicated that non-sport-related HT was a significant risk factor for PD (OR 2.4, 95% CI 1.3–4.5; *p* < 0.01); conversely, sport-related HT was not significantly associated.

### Head trauma and clinical-biochemical profile

TS did not correlate with clinical parameters (UPDRSIII, H&Y, MMSE, NMSS, LEDD) in PD patients. Likewise, these parameters did not differ between Tr + and Tr− patients, neither in models corrected for covariates (age, sex, disease duration, LEDD).

**Fig. 2:**
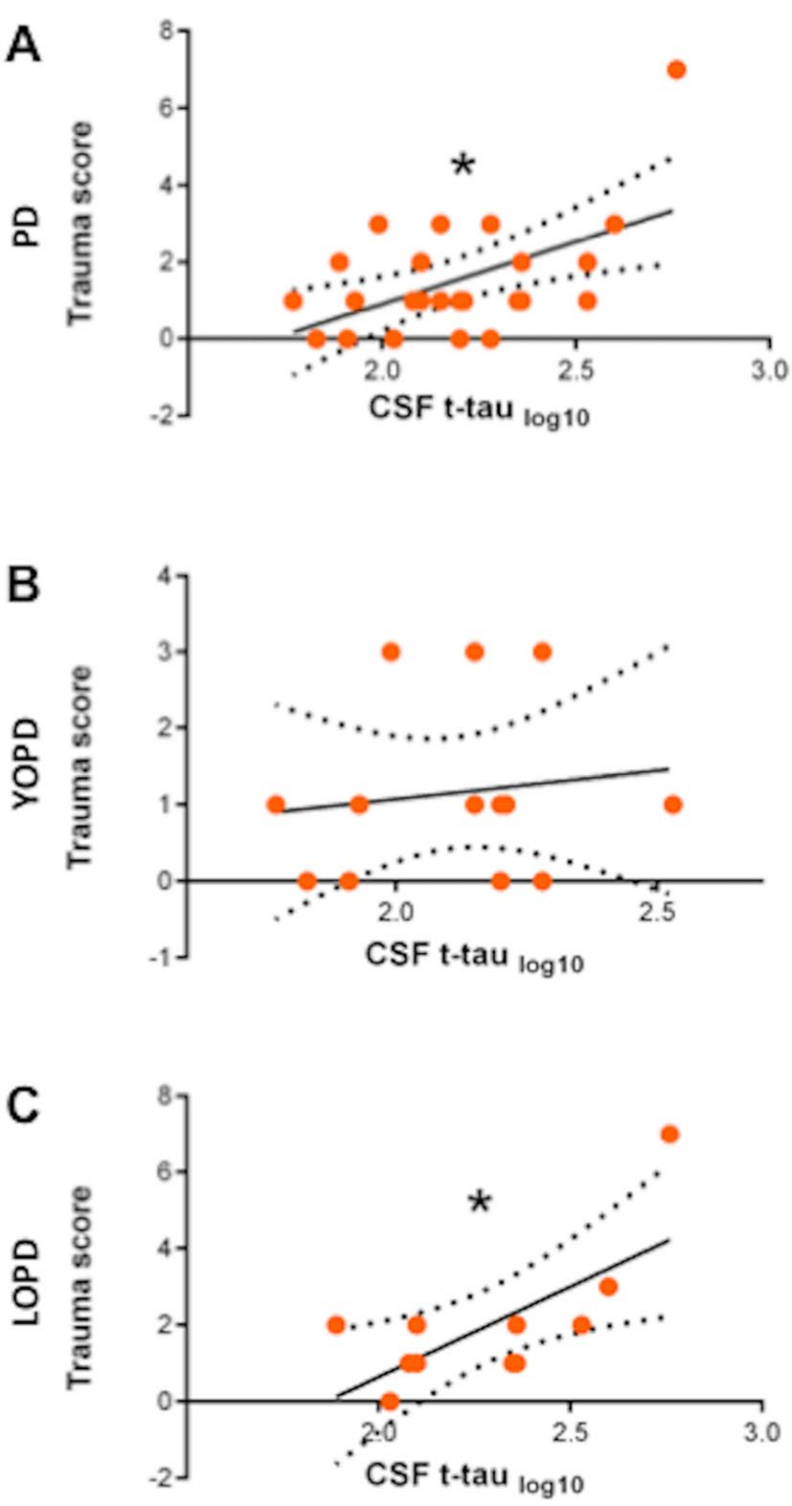
Dot plots graphing simple linear regression between TS and CSF t-tau (log_10_ transformed value) in PD (**A**), YOPD (**B**), and LOPD (**C**). Asterisks indicate statistical significance (*p* < 0.05)

TS had a direct association with CSF t-tau [*F*(1,22) = 8.9, *p* = 0.007, *R*^2^ = 0.29. *T* = 2.98, *p* = 0.007], even confirmed into a model including age, sex, and disease duration as covariates [*F*(4,18) = 2.7, *p* = 0.05, *R*^2^ = 0.38. *T* = 2.9, *p* = 0.01] (Fig. 2). No further associations with other CSF biomarkers (α-syn, Aβ42, t-tau, p-tau, lactate, AR) resulted. CSF biomarker levels did not differ between Tr + and Tr− patients, even when age, sex, and disease duration were included as covariates.

### Head trauma and young-onset PD

Age at PD onset was significantly younger in Tr + patients (56.6 ± 10.5 years) than Tr− patients (63.3 ± 11.7) [*F*(1,90) = 7.6, *p* = 0.007]. TS, however, did not correlate with age at onset, nor differ between YOPD and LOPD.

The proportion of Tr + patients was significantly higher in the YOPD group (80.6%) than LOPD (59%) (*p* < 0.05) (Fig. 1). Positive HT history was a significant risk factor for YOPD (OR 2.9, 95% CI 1.03–8.1; *p* < 0.05).

Minor HTs (without consciousness impairment) were 58% in YOPD and 67% in LOPD (*p* = NS). Also, the prevalence of TBI did not significantly differ between YOPD (42%) and LOPD (33%). TBI with altered mental status was reported in 29% of YOPD patients and 26% of LOPD (*p* = NS); TBI with loss of consciousness was reported in 32% in YOPD patients and 21% of LOPD (*p* = NS).

Sport-related HT was more frequent in YOPD (22.6%) than LOPD (4.9%) (*p* = 0.01), and was a significant risk factor for YOPD (OR 5.6, 95% CI 1.3–23.7; *p* = 0.018). Non-sport-related HT occurred with similar frequency in YOPD (74.2%) and LOPD (57.4%) (*p* = NS). Accordingly, binomial logistic regression found no significant association between non-sport HT and risk for YOPD.

In YOPD group, no significant associations resulted between TS and clinical parameters or CSF biomarkers, even when relevant covariates were included. In LOPD group, the only significant correlation was the direct association between TS and CSF t-tau [*F*(1,8) = 8.3, *p* = 0.02, *R*^2^ = 0.45. *T* = 2.9, *p* = 0.02], which was not confirmed by the model adjusted for age, sex and disease duration (Fig. 2).

In YOPD group, CSF t-tau level was lower than LOPD group [*F*(1,21) = 4.16, *p* = 0.05]; no other relevant differences resulted between YOPD and LOPD groups in clinical-biochemical parameters.

### Sporting activities

Because of relevance of sport-related HT, the contribution of sport practice to risk of HT and PD has been specifically assessed.

Individuals that practiced sports were more numerous among Tr + subjects than Tr− (54.9% vs 38.4%, *p* = 0.03). However, rates of sport participation were equivalent between PD and controls, Tr + patients and Tr− patients, YOPD and LOPD. TS did not differ between sportive individuals and non-sportive in the whole population, nor in PD and controls separately.

Practiced sports did not differ in Tr + vs Tr− subjects, PD vs controls, Tr + patients vs Tr− patients, YOPD vs LOPD (Table 2).

**Table 2:** Participation to different types of sport per group

	PD (%)	CTL (%)	Significance	YOPD (%)	LOPD (%)	Significance
Any sport	53.2	40.0	ns	64.5	49.2	ns
Team sports	39.1	21.4	ns	37.9	40	ns
Water sports	5.8	9.5		10.3	2.5	
Individual sports	10.1	9.5		6.9	12.5	
Athletics/Gymnastics/Running	23.2	45.2		24.1	22.5	
Contact sports	5.8	4.8		10.3	2.5	
Other sports	15.9	9.5		10.3	20	

Abbreviations are spelled in the text

Number of years practicing sport did not correlate with TS in the whole population, nor in PD and controls, separately.

**Fig. 3:**
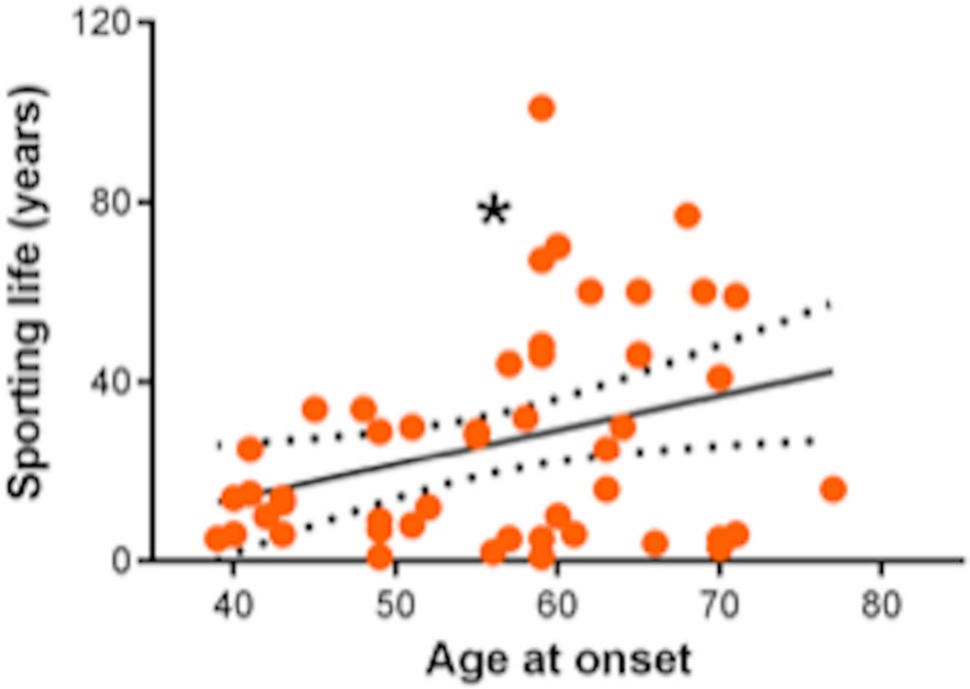
Dot plot graphing simple linear regression between age at onset (in years) and duration of sporting life (in years). Asterisk indicates statistical significance (*p* < 0.05)

Number of years practicing sport did not differ in Tr + vs Tr− subjects, PD vs controls, Tr + patients vs Tr− patients, YOPD vs LOPD. However, duration of sport practice was directly associated with age at PD onset [*F*(1,47) = 5.6, *p* = 0.02, *R*^2^ = 0.107; *T* = 2.237, *p* = 0.02] (Fig. 3). Accordingly, longer sport practice reduced the risk for YOPD (OR 0.96, 95% CI 0.927–0.996; *p* = 0.02).

Number of years practicing sport did not correlate with other clinical parameters and CSF biomarkers in PD group, nor in YOPD and LOPD groups separately.

## Discussion

In this study, we applied the BISQ to systematically assess and characterize HT history in a cohort of PD patients to estimate the disease risk due to cumulative lifetime head injuries, define the impact of HT on the age of PD onset, and explore the correlations with the clinical-biochemical profile of patients.

Occurrence and frequency of head injuries resulted higher in PD patients than controls. HTs, thus, increased the risk for PD proportionally to their number, according with previous data showing the pathogenic role of HTs in PD (Jafari et al. 2013; Nicoletti et al. 2017). In fact, HTs can affect in a “dose-dependent” manner (Gardner et al. 2015) the biological pathways involved in PD, including energy metabolism, proteinopathy, neuroinflammation, and blood–brain-barrier (BBB) integrity (Delic et al. 2020), favoring the disease appearance (Gardner et al. 2015).

Patients with HT history also presented an earlier PD onset, being in risk for YOPD. YOPD patients have distinctive clinical-biochemical features compared to those with LOPD (Schirinzi et al. 2020b) and, even in the absence of known genetic variants (as in our cohort), they are considered a genetically vulnerable category in which some environmental factor leads to disease onset (Laperle et al. 2020). Therefore, HT emerged as one of the culprit risk factor for YOPD, confirming findings of a single previous work (Tsai et al. 2002).

Severity of HTs, here, did non differ between YOPD and LOPD, suggesting that subclinical and milder HTs (Gardner et al. 2018; Wu et al. 2020) are sufficient to precipitate the vulnerability to YOPD. However, as expected (Gardner et al. 2015), in the whole PD group, HTs with loss of consciousness (TBI) were more frequent than in controls, increasing the risk for the disease overall.

PD patients may have had HT either during sports or non-sportive activities, but only the latter events were significantly associated with the disease. Sport-related HT, instead, prevailed in YOPD group compared to LOPD, suggesting that repetitive exposure to subclinical HT during sport may be a specific risk factor for YOPD. These data actually agree with reports indicating professional athletes as prone to neurodegenerative diseases, especially with early or young onset, as a consequence of the repeated HTs (Lehman 2013; Mackay et al. 2019). However, in our population of non-professional athletes, neither any sport in particular nor the duration of sport practice led to increased risk for PD, probably because HTs occurred by chance rather than because of intensive training or contacts.

Conversely, we noticed that a longer duration of sport practice could delay the age of PD onset, supporting the robust evidence on the neuroprotective action of physical activity in PD (Shih et al. 2016; Alwardat et al. 2019; Schirinzi et al. 2020a) and highlighting the role of prolonged sport activity as a protective factor for YOPD. Actually, these findings do not contradict the risks associated with sport-related HT, but, instead, follow the evidence obtained in ALS, whose risk in athletes has been linked to the frequency of head and cervical traumas rather than to the practice of sport itself (Blecher et al. 2019).

To further elucidate the relationship between HT and PD, we investigated correlations with clinical parameters and CSF biomarkers of neurodegeneration. HT history did not affect main clinical disturbances of the disease (both motor and non-motor), neither in the PD whole group, nor in YOPD and LOPD subgroups, even when fundamental covariates were considered into the statistical models. Contrariwise, the TS, namely the individual number of HTs, exhibited a direct and independent association with CSF t-tau levels, which may offer some insight on the potential mechanisms underlying the link between HT and PD.

CSF t-tau is a well-known index of neuronal and axonal degeneration, which typically increases in several brain disorders with neuronal loss or overt degeneration (Molinuevo et al. 2018; Abu-Rumeileh et al. 2020). CSF t-tau levels in PD patients have varied across studies, with most reporting values that are either lower or similar to controls (Parnetti et al. 2019). However, in PD patients, the increase of CSF t-tau content is believed to predict the spreading of cerebral tau pathology (Gomperts et al. 2016; Irwin et al. 2018), the disruption of brain connectivity, and the degeneration of substantia nigra (Zhang et al. 2016; Abbasi et al. 2018). CSF t-tau levels, in turn, can also increase in subjects who have sustained repeated HTs, correlating with structural abnormalities of cerebral white matter, as a recent study on former athletes with multiple brain concussions demonstrated (Taghdiri et al. 2019). The association between TS and CSF t-tau levels in PD patients may thus reflect neuropathological change associated with HTs, in which tau protein is involved with a subsequent, proportional increase of its CSF content. Since tauopathy is central in neurodegenerative processes, either in those following traumatic brain injury or in those related to PD (Delic et al. 2020), current findings support the notion that the risk of PD due to HT may result from a tau-mediated neurodegenerative mechanism, which is consistent with data showing the centrality of tau pathology in neuropsychiatric disturbances of patients with traumatic brain injury (Takahata et al. 2019).

However, aging seems to influence the association between HTs and tauopathy. In fact, the correlation resulted in the whole PD group and in LOPD subgroup, but not in YOPD patients. Since post-concussive increase of CSF t-tau augments with age (Taghdiri et al. 2019), and CSF t-tau levels could be higher in LOPD than in YOPD patients (Schirinzi et al. 2020b), we may presume that HTs contribute to YOPD by precipitating the genetic vulnerability through mechanisms different from tau-mediation. Overall, the absence of correlations between HT and other CSF biomarkers suggests the prominent and selective role of tau protein in neuropathological events following traumatic injuries.

There are several limitations to be acknowledged in this study, including the sample size, the retrospective design with associated biases, the exclusion of known protective factors in PD risk estimation (e.g. coffee consumption), and the absence of a control group for the correlation analysis. Conversely, the use of a comprehensive and structured tool for lifetime HT history assessment represents an important advancement over prior studies that investigated only injuries with specific characteristics or sustained during the timeframe for which medical records were available.

Besides the limitations, this study provided additional evidence on HT as “dose-dependent” risk factor for PD, and disclosed in vivo the involvement of tauopathy in such association. Lastly, we found that HT was a specific risk factor for YOPD. In our interpretation, HT might contribute to YOPD through molecular pathways other than tauopathy, supporting the hypothesis that YOPD has distinctive features from typical, later onset PD. HTs associated to YOPD mostly occurred during sport practice; on the other hand, the longer sporting life delayed PD onset, thus appearing YOPD patients particularly sensitive to environmental factors, either when causative or protective for the disease.

These preliminary findings definitely need to be validated in further, larger studies, with prospective design. However, we drew attention on HT, even in the absence of clinical TBI, as risk factor for PD, and particularly for YOPD; we also highlighted the associated role of tauopathy, providing critical insights into potential therapeutic or preventive targets.

## Data Availability

Available upon reasonable request.
